# Avian Influenza Virus with Hemagglutinin-Neuraminidase Combination H3N6, Isolated from a Domestic Pigeon in Guangxi, Southern China

**DOI:** 10.1128/genomeA.01537-14

**Published:** 2015-02-05

**Authors:** Tingting Liu, Zhixun Xie, Guoli Wang, Degui Song, Li Huang, Zhiqing Xie, Xianweng Deng, Sisi Luo, Jiaoling Huang, Tingting Zeng

**Affiliations:** a College of Life Science, Guangxi Normal University, Guilin, Guangxi Province, China; b Guangxi Key Laboratory of Animal Vaccines and Diagnostics, Guangxi Veterinary Research Institute, Nanning, Guangxi Province, China; c Guangxi Animal Husbandry Service, Nanning, Guangxi Province, China

## Abstract

The H3 subtype of avian influenza virus can provide genes for human influenza virus through gene reassortment, which has raised great concerns about its potential threat to human health. An H3N6 subtype of avian influenza virus was isolated from Guangxi Province, China, in 2009. All eight gene segments of the strain were sequenced. The sequence analysis indicated that this H3N6 virus was a nature reassortant virus. The genome sequences now can be used to understand the epidemiological and molecular characteristics of the H3N6 influenza virus in southern China.

## GENOME ANNOUNCEMENT

Avian influenza virus (AIV) belongs to the type A influenza viruses, which infect many avian species (1). At present, there are 18 hemagglutinin (HA) and 11 neuraminidase (NA) subtypes of AIV based on the antigenic differences of the HA and NA proteins, which are surface glycoproteins on the viral envelope (2, 3). The H3 subtype of AIV belongs to the low-pathogenic AIVs (LPAIVs) and is one of predominant subtypes among the LPAIVs (4, 5). Researchers have shown that the H3 subtype AIV has a high separation rate in poultry, and it may have the ability to cross the species barrier to infect humans through gene reassortment (6–8). In addition, previous studies have demonstrated that some novel H3N6 subtype viruses were reassortants between highly pathogenic H7 and H5 viruses isolated in Eurasia (9, 10), thus signifying the importance of enhancing the surveillance of the H3N6 subtype AIV.

An H3N6 subtype AIV was isolated from a pigeon in a live poultry market in Guangxi, China, in May 2009 and named A/pigeon/Guangxi/020P/2009 (H3N6). In this study, we amplified the full genes by reverse transcription-PCR using AIV universal primers (11, 12). The amplified products were purified and cloned into the pMD-18T vector (TaKaRa, Dalian, China) and sequenced (TaKaRa). The sequences were assembled using the SeqMan program and manually edited to generate the final full-length genome sequence.

The complete genome of this H3N6 strain consisted of eight gene segments of polymerase basic 2 (PB2), PB1, polymerase acidic (PA), HA, nucleoprotein (NP), NA, matrix (M), and nonstructural (NS) genes. The full lengths of these segments were 2,341, 2,341, 2,233, 1,765, 1,565, 1,464, 1,027, and 890 nucleotides, respectively. Those eight genes encoded proteins with the following amino acid lengths: 759 (PB2), 757 (PB1), 716 (PA), 566 (HA), 498 (NP), 470 (NA), 252 (M1), 97 (M2), 230 (NS1), and 121 (NS2). The amino acid sequence at the cleavage site (positions 340 to 348) of the HA molecule was PEKQTR↓GLF, with one basic amino acid, which is characteristic of low-pathogenic AIV. The amino acid residues at the receptor binding site in the HA protein were Q226 and G228, which are different from L226 and S228 in the H3 subtype of human influenza viruses, which preferentially bind to an avian-origin receptor. An analysis of potential glycosylation sites revealed that there were 6 potential *N*-linked glycosylation sites in the HA protein (positions 24, 38, 54, 181, 301, and 499), while there were 8 in NA (positions 51, 54, 62, 67, 70, 86, 146, and 402). In addition, the PB2 protein identified in this isolate contained E627 and D701, which indicated that the virus was of avian origin (13, 14).

The analysis of the sequence also indicated that the nucleotide sequences of both the HA and NA genes of this H3N6 strain belong to the Eurasian lineage. Also, its other internal genes are closely related to H3N8, H4N6, H6N2, H3N2, and H4N2 subtype AIVs, which suggests that this H3N6 strain went through extensive reassortment with different subtypes of influenza viruses. The genome information of the isolated virus revealed in this study can now be used for conducting an epidemiological investigation on the H3N6 subtype of AIV in China.

### Nucleotide sequence accession numbers.

The genome sequence of A/pigeon/Guangxi/020P/2009(H3N6) has been deposited in GenBank under the accession numbers KM186122 to KM186129.
